# “Do my Roma and non-Roma patients need different care?” A brief step-by-step guideline for clinical practitioners

**DOI:** 10.1007/s00038-019-01246-9

**Published:** 2019-05-21

**Authors:** Andrej Belak, Andrea Madarasova Geckova, Jitse P. van Dijk, Sijmen A. Reijneveld

**Affiliations:** 1 Department of Health Psychology, Faculty of Medicine, P.J. Safarik University, Trieda SNP 1, 040 11 Kosice, Slovakia; 2 Graduate School Kosice Institute for Society and Health, Faculty of Medicine, P.J. Safarik University, Kosice, Slovakia; 3 Department of Community and Occupational Medicine, University Medical Center Groningen, University of Groningen, Groningen, The Netherlands; 4 Department of General Anthropology, Faculty of Humanities, Charles University, Prague, Czech Republic; 5 Olomouc University Society and Health Institute, Palacky University Olomouc, Olomouc, Czech Republic

## Introduction

As researchers regularly publishing on Roma health in Slovakia and beyond, we often get approached by alerted clinical practitioners who treat Roma patients. Usually, they contact us with the impression that their Roma and their non-Roma patients have significantly different symptoms, morbidity or care outcomes and question how they could diversify and tailor their care accordingly. Fellow researchers elsewhere in Central and Eastern Europe (CEE) are likely to face similar requests for help (Cook et al. 2013; Crowe 2007).

Here, we offer a step-by-step guideline for further investigation and accommodation of such seeming differences. However, as the practitioners approaching us themselves most often suspect major genetic influences, we will start with brief reiterations of why genes are the least and social determinants the most reasonable suspects to begin investigation with in this and in similar cases.

## Why should genes come last?

To expect major genetic influences behind ethnic health disparities is unreasonable according to both the principles of population genetics and related evidence on social health disparities. Any population genetically more predisposed for a range of health problems should have been previously selected systematically, whether naturally or intentionally, for the unhealthy predispositions (Haydon 2007; Yudell et al. 2016). Such logically tense proposition seems highly unlikely also in the light of evidence on inter- and intra-group patterns in health status not corresponding to known patterns in genetic variability (Diez Roux 2012; WHO 2013; Yudell et al. 2016).

Accordingly, and alike for other ethnic health disparities (Diez Roux 2012; Dressler et al. 2005), the insignificance of genetic influences behind poor Roma health status has been confirmed empirically. The only genetic susceptibilities identified in Roma are higher frequencies of a handful of gene alleles causing rare diseases, peaking in some localities due to total social (reproductive) segregation from neighbouring populations (Diószegi et al. 2017; Fiatal et al. 2016; Kalaydjieva et al. 2001; Martinez-Cruz et al. 2016). Let us emphasize that this is despite a previous disproportionate focus of research specifically on possible genetic influences (Hajioff and McKee 2000; Zeman et al. 2003).

## Why should social determinants come first?

According to epidemiological theory, social health disparities are almost always established and maintained socially. There are many other common ways for human bodies to get damaged beyond the above-discussed genetic susceptibilities to diseases, ranging from unfavourable material living conditions and risky health-related practices to stress. Health disparities between social groups are typically determined through socially maintained distinct combinations and the interplay of all such exposures over the life course (Diez Roux 2012; Krieger 2011; WHO 2010).

Like for other major ethnic health disparities (Bailey et al. 2017; Bhopal 2015; Dressler 2010), empirical evidence on CEE Roma health disparity fits the epidemiological theory well. Over the last ten years, research has shown that most of this disparity, too, can be explained by socially disadvantaged segments of the worse-off population disproportionately facing a wide range of environmental, behavioural, psychological and care-related exposures over the life course (e.g. Arora et al. 2016; Cook et al. 2013; EUC 2014; Geckova et al. 2014; Masseria et al. 2010).

Thus, any clinical discrepancies between Roma and non-Roma also most likely originate from, are maintained by, and can be alleviated via adjustments of social processes supporting unequal exposures over the life course. Different exposures can and do get embodied across ethnic divides; they then become biology (Bailey et al. 2017; Bhopal 2015; Gravlee 2009). However, as such differences only present the results of social differences, the tackling of their adverse clinical outcomes should start with assessing the underlying exposures, related social processes and their social root causes such as racism within and beyond healthcare (Feagin and Bennefield 2014; Phelan and Link 2015).

## Guideline

**Fig. 1:**
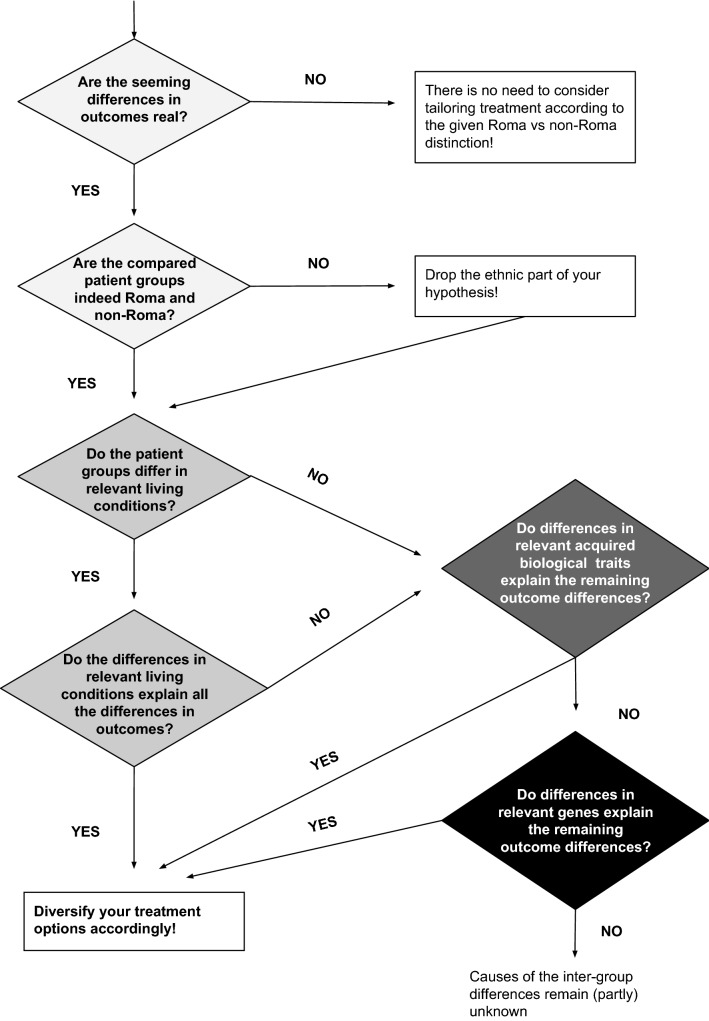
A step-by-step guideline for dealing with apparent differences in Roma and non-Roma patients

Drawing on the above and related experience, we suggest the following approach (see also Fig. 1):

1. Are the seeming differences in outcomes real?
  Do the studied differences remain statistically significant after adjustments for likely differences in the demographics of the different populations the compared patient groups are supposed to represent?
  Until this question can be answered “yes”; there is no need to consider tailoring treatment according the given Roma and non-Roma distinction criteria
2. Are the compared groups indeed Roma and non-Roma?
  Do all patients in both groups agree to being labelled as such according to a set of unambiguous criteria? Ethnicity labelling can be constructed and contested in many ways by both those labelled and those labelling (Janka et al. 2018; Ladányi and Szelényi 2001).
  If “no”, drop the ethnic part of the hypothesis but continue with the next step (3).
  If “yes”, specify the ethnic aspect of your hypothesis (e.g. What kind of Roma?) according to the identification criteria used and continue with the next step (3).
3. Do the patient groups differ in relevant living conditions?
  Do available databases or follow-up communication with the patients indicate that the compared groups face living conditions that are distinct in aspects which might relate to the studied differences in outcomes?
  If “yes”, continue with the investigation of possible causes related to living conditions (4).
  If “no”, continue with the investigation of possible acquired biological causes (5).
4. Do the differences in relevant living conditions explain all the differences in outcomes?
  Do all the studied differences in outcomes between the compared patient groups disappear after statistical adjustments for the differences in relevant aspects of the groups’ living conditions?
  If “yes”, try to develop and include among treatment options treatment plans that also account for the found influences of living conditions (e.g. Bourgois et al. 2017)
  If “no”, continue with the investigation of possible acquired biological causes (5).
5. Do differences in relevant acquired biological traits explain the remaining outcome differences?
  Does additional clinical testing show that the compared groups might have acquired different biological traits, which might relate to the studied outcomes?
  If “yes”, try to develop and include among treatment options treatment plans that account for the found influences of acquired biological differences
  If “no”, continue with the investigation of possible genetic causes (6).
6. Do differences in relevant genes explain the remaining outcome differences?
  Does additional clinical testing show that the compared groups have genetic variants which might relate to the studied outcomes?
  If “yes”, try to develop and include among treatment options treatment plans that account for the found influences of genes
  If “no”, you were not able to identify some of the causes behind the existing differences.

## Conclusion

We have herein proposed and justified a step-by-step guideline for dealing with apparent clinical differences in Roma and non-Roma patient groups. The guideline recommends that clinical practitioners facing such differences take a specific route. This route starts with assessing the statistical significance and representativeness of the difference through clarification and legitimization of ethnicity criteria, then goes on to assessment of differences in relevant living conditions and only arrives at assessing biological differences if refuting the preceding.
